# Changes in Tobacco Quitlines in the United States, 2005-2006

**Published:** 2010-02-15

**Authors:** Paula A. Keller, Annamaria Feltracco, Linda A. Bailey, Zhanhai Li, Jeff Niederdeppe, Timothy B. Baker, Michael C. Fiore

**Affiliations:** University of Wisconsin Center for Tobacco Research and Intervention; Feltracco Consulting, Waterford, Ontario, Canada. At the time of the study, Ms Feltracco was affiliated with the North American Quitline Consortium, Phoenix, Arizona; North American Quitline Consortium, Phoenix, Arizona; University of Wisconsin, Madison, Wisconsin; Cornell University, Ithaca, New York. At the time of the study, Dr Niederdeppe was affiliated with the University of Wisconsin, Madison, Wisconsin; University of Wisconsin Center for Tobacco Research and Intervention, Madison, Wisconsin; University of Wisconsin Center for Tobacco Research and Intervention, Madison, Wisconsin

## Abstract

**Introduction:**

Telephone quitlines are an effective way to provide evidence-based tobacco dependence treatment services at the population level. Information about what services quitlines offer and how those services are used may improve their reach to the smoking population.

**Methods:**

The North American Quitline Consortium surveyed state quitlines in 2005 and 2006 to get information about quitline services, funding, and use. We report changes between 2005 and 2006.

**Results:**

By 2006, all 50 states, the District of Columbia, and Puerto Rico had quitlines, and annual mean reach was approximately 1% of US adult smokers (aged 18 years or older). Significant increases were seen in mean quitline reach, mean per capita funding for quitline services, and provision of free cessation medications; otherwise, few changes were seen in quitline services.

**Conclusions:**

Quitlines have the potential to serve a large percentage of smokers. Between 2005 and 2006, gains in the number, reach, and per capita funding for quitline services in the United States were seen. Although this represents progress, further research and investment to optimize quitline service delivery and reach are required for quitlines to fulfill their potential of improving the health of the American population.

## Introduction

The effectiveness of telephone quitlines for smoking cessation is well documented (1-5). The 2008 Public Health Service clinical practice guideline update found that quitline counseling was more than 1.5 times as effective as minimal interventions or self-help materials (5). This finding was consistent with a Cochrane analysis that found multisession quitline counseling to be more effective than self-help materials or brief single-session counseling (4).

California was the first state to implement a publicly funded quitline, in 1992. As the evidence base expanded, the number of quitlines increased rapidly. By the end of 2006, all 50 states, the District of Columbia, and Puerto Rico had implemented publicly funded quitlines (6). Our objective was to evaluate changes in US quitlines by focusing on changes in reach, service characteristics, and funding. We report on the use of Centers for Disease Control and Prevention (CDC) funds to develop or enhance quitline services. The findings of this research have the potential to inform quitline service providers, state tobacco control programs, and policy makers how to improve quitline treatment services.

## Methods

We analyzed data from the 2005 and 2006 North American Quitline Consortium (NAQC) surveys of state and provincial quitlines. The 2005 and 2006 NAQC surveys were distributed to state and provincial tobacco control programs and quitline vendors by e-mail. In 2005, respondents completed a Microsoft Word version of the survey and returned it to the University of California at San Diego for data entry and cleaning (7). In 2006, SurveyMonkey was used for data collection (8). Staff from NAQC and the University of Wisconsin Center for Tobacco Research and Intervention reviewed all responses for completeness and clarity; NAQC staff contacted states and provinces to clarify responses. Only US states and territories were included in the analysis. Missing responses for particular variables were excluded from analysis of those variables only.

We estimated per capita cost of quitlines by using US census data (9,10). Cost per adult smoker and reach were estimated by using data on adult smoking prevalence from the Behavioral Risk Factor Surveillance System and from the US census (9-12). Reach was defined as the total number of calls to the quitline divided by the estimated number of adult smokers in the state.

We used SPSS version 15 (SPSS, Inc, Chicago, Illinois) and Microsoft Excel (Microsoft Corporation, Redmond, Washington) for analysis. We compared changes by using the McNemar χ^2^ test for categorical data and a *t* test for paired samples of continuous data. Continuous data with a nonnormal distribution were log-transformed to approximate normality before analysis. For budget data, we removed values outside of 3 standard deviations from the mean from the analyses. Results were considered significant at *P* ≤ .05.

The University of Wisconsin Health Sciences Minimal Risk Institutional Review Board determined the study to be exempt from review.

## Results

The survey response rate was 100% in both 2005 and 2006. The number of states or territories with quitlines grew from 50 to 52 during this time, and significant increases in the mean number of quitline calls (5,866 vs 6,384, *P* = .01) and in mean quitline reach (0.9% vs 1.0%, *P* = .05) were seen (Table 1). We found no significant differences in any of the types of quitline counseling provided between 2005 and 2006 (minimal/brief intervention, single-session counseling, multisession reactive counseling, or multisession proactive counseling).

More than half of US quitlines had eligibility criteria for receipt of service. Among quitlines with such criteria, we noted a significant difference in the number of quitlines with age as an eligibility criterion (13 vs 19, *P* = .05). We found no significant changes in the number of quitlines reporting readiness to quit, insurance status, and being a member of a special population as eligibility criteria.

Nearly all US quitline counseling protocols dictated the number of counseling sessions to be provided. No significant differences were seen in the mean number of sessions dictated by the protocol, the mean length of the first counseling session, or the mean length of the follow-up counseling session. Quitlines reported significant increases for both the number of quitlines offering free cessation medications to callers (18 vs 24, *P* = .05) and for specific types of medications provided (Table 2). Most quitlines also reported having eligibility criteria for a caller to receive free medications.

Nearly all quitlines provided counseling and materials in English and Spanish. Few quitlines provided counseling and materials in other languages (eg, Chinese, Mandarin, Vietnamese). Almost all quitlines had some specialized counseling protocols in place (Table 3), and most used specialized materials. Quitlines also routinely provided materials to callers who were not tobacco users (eg, health professionals, proxy callers) and used the Internet to provide information and services, but changes in these characteristics from 2005 to 2006 were not significant.

Both quitline service providers and the types of organizations that fund quitlines varied little between 2005 and 2006. We saw no significant changes in the percentage of nonprofit or government organizations, health care or research institutions, and for-profit organizations providing quitline services and the percentage of states reporting multiple service providers. Although the percentage of states reporting any state funding for their quitline increased, this change was not significant.

Between 2005 and 2006, significant increases were seen in mean quitline service budgets ($836,858 vs $887,603, *P* = .04), mean per capita service budgets ($0.26 vs $0.29, *P* = .03), and mean per adult smoker service budgets ($1.67 vs $1.93, *P* = .02) (Table 4). We observed no significant changes with regard to promotion budgets between 2005 and 2006.

Most US quitlines reported that their state or territory had applied for supplemental funding from CDC for their quitline. The 2 most commonly reported ways CDC funds were used in both 2005 and 2006 were to expand outreach to specific populations (57% vs 50%) and to expand marketing efforts (51% vs 52%), but changes in how states used the funding from CDC were not significant.

## Discussion

By 2006, US residents in the 50 states, Washington, DC, and Puerto Rico had access to quitline services, in contrast to 2004, when residents of only 38 states had access (13).

We found a significant increase in the percentage of smokers reached by quitlines between 2005 and 2006, although the percentage reached was modest (0.9% in 2005 and 1.0% in 2006). This finding is consistent with previous analyses of NAQC data that estimated reach at approximately 1% (7,13). Other researchers have published examples of state tobacco control efforts that have resulted in rates of 2% to more than 6% (14-16). Even these promising efforts fall short of the goal of reaching 8% of smokers annually recommended by CDC in *Best Practices for Comprehensive Tobacco Control Programs — 2007* (17). Additional innovations to help quitlines increase reach are needed, as are data to evaluate future changes in reach.

The cost for quitline services and promotion remains modest. We found a significant increase in mean per capita quitline services budgets. We also noted a slight increase in mean per capita quitline promotion budgets, although this change was not significant. Our analysis of earlier NAQC survey data found the median per capita quitline services budget was $0.14 and the median per capita promotion budget was $0.09 (13). Increases in the number of states providing free cessation medications to callers may explain the change in service budgets; some states have reported using free cessation medications as a quitline promotional strategy (15). Nevertheless, this investment to provide and promote population-wide tobacco cessation services is modest, particularly in light of the amount of revenue generated by state tobacco excise taxes and funds from the Master Settlement Agreement, and we encourage states to consider enhancing their investment in quitline services.

Quitlines reported few changes in the types of counseling offered. However, the number of quitlines offering free cessation medications increased significantly, from 36% to 46%. In contrast, in 2004, only 21% of quitlines offered free cessation medications (13). This change in quitline treatment services is consistent with the 2008 Public Health Service guideline update, which recommends both quitline counseling alone and quitline counseling with medications as efficacious cessation services (5). It is also consistent with CDC's *Best Practices for Comprehensive Tobacco Control Programs*, which includes both quitline counseling and nicotine replacement therapy in its recommended budget estimates for state cessation programs (17). Ongoing study of the types of quitline services offered is warranted, both to track changes and to assess whether the recommendations for quitline services outlined by CDC are implemented.

Changes in the types of funds available for quitlines fluctuated. Additional research into how quitlines are funded is warranted, both to monitor changes and to identify factors that may sustain and increase financial support of quitline services.

This study has some limitations. First, the data were incomplete for some responses on both the 2005 and 2006 NAQC surveys. Second, only the 2005 and 2006 NAQC survey data were used because of substantial changes in the survey between 2004 and 2005. Two years of data may not be sufficient to show changes. Third, the NAQC survey includes only state and provincial quitlines; quitlines operated by health insurers or employers are not part of the survey. Finally, our definition of reach did not exclude people who contacted the quitline more than once, nor did it include an estimate of smokeless tobacco users in the US population, potentially inflating the reach estimate. Approximately 3% of US adults use smokeless tobacco. Of those, 39% report past-month cigarette use (18). A strength of this study is that we are able to report on all state quitlines. However, the magnitude of changes in some of the data may appear dramatic yet not be significant given the small number of state quitlines.

Quitlines are an effective way to provide evidence-based tobacco dependence treatment services at the population level. Significant increases were seen in quitline reach, mean per capita funding for quitline services, and provision of free cessation medications. Further research and investment to optimize quitline service delivery and reach are required for quitlines to fulfill their potential of improving population health (19).

## Acknowledgments

Analyses were funded by grant no. 053133 from the Substance Abuse Policy Research Program, Robert Wood Johnson Foundation. The funders of the NAQC survey include the American Legacy Foundation (grant no. 6009), the Academy for Educational Development (contract no. 3303-02-S-01), and the American Cancer Society (grant no. 8911029).

We thank Eric Beyer for his assistance with this manuscript.

Disclosures: Dr Baker has served as an investigator on research projects sponsored by pharmaceutical companies including Pfizer, Glaxo Wellcome, Sanofi, and Nabi Biopharmaceuticals. Over the last 3 years, Dr Fiore has served as an investigator in research studies at the University of Wisconsin that were funded by Pfizer, GlaxoSmithKline, and Nabi Biopharmaceuticals. In 1998, the University of Wisconsin appointed Dr Fiore to a named chair funded by a gift to the university from Glaxo Wellcome.

## Figures and Tables

**Table 1:** Changes in Number of Calls and Reach for State Tobacco Quitlines, North American Quitline Consortium Survey, 2005-2006

Variable	2005 Mean (SD)	**n** a	2006 Mean (SD)	**n** a	*P* Value
No. of quitline calls^b^	5,866 (4)	29	6,384 (5)	37	.01
Reach^c^	0.9% (1.2%)	40	1.0% (1.3%)	47	.05

a States that did not report the number of quitline calls were excluded from this analysis.

b Data were log-transformed to approximate normality.

c Reach: total quitline calls divided by the number of adult smokers aged 18 years or older.

**Table 2:** Changes in Provision of Free Cessation Medications by State Tobacco Quitlines, North American Quitline Consortium Survey, 2005-2006

**Type of Medication^a^**	2005 No. (%) (N = 50)	2006 No. (%) (N = 52)	*P* Value^b^
Any	18 (36)	24 (46)	.05

	**2005 No. (%) (n = 18)**	**2006 No. (%) (n = 24)**	

Patch	17 (94)	24 (100)	NC^c^
Gum	11 (61)	23 (96)	.03
Lozenge	2 (11)	20 (83)	<.001
Bupropion	0	18 (75)	NC^d^
Other	0	15 (63)	NC^d^

Abbreviation: NC, not calculated.

a Multiple responses were permitted.

b Calculated by using McNemar χ^2^ test.

c *P* value not calculated because the final value was 100%.

d *P* value not calculated because the initial value was 0.

**Table 3:** Changes in the Provision of Specialized Counseling Protocols by State Tobacco Quitlines, North American Quitline Consortium Survey, 2005-2006

**Population**	2005 No. (%) (n = 46)^a^	2006 No. (%) (n = 47)^a^	*P* Value^b^
Pregnant women	44 (96)	46 (98)	.99
Children aged 12-17 y	23 (50)	27 (57)	.47
Adults aged 18-24 y	8 (17)	3 (6)	.05
Older tobacco users (≥55 y)	5 (11)	3 (6)	.32
Smokeless tobacco users	36 (78)	39 (83)	.74
Racial/ethnic minority populations	26 (57)	23 (49)	.16
People who are lesbian, gay, bisexual, transgendered	6 (13)	0	NC^c^
People with chronic mental illness/psychiatric conditions	5 (11)	1 (2)	.05
People who have multiple addictions: tobacco and alcohol or other drugs	8 (17)	2 (4)	.01
Other	5 (11)	11 (23)	.08

Abbreviation: NC, not calculated.

a Multiple responses were permitted.

b Calculated by using McNemar χ^2^ test.

c *P* value not calculated because the final value was 0.

**Table 4:** Changes in Budgets for State Tobacco Quitlines, National American Quitline Consortium Survey, 2005-2006

**Cost**	2005 Mean, $ (SD)	No. of Quitlines	2006 Mean, $ (SD)	No. of Quitlines	*P* Value^a^
**Service**					
Total	836,858 (847,178)	43	887,603 (1,059,554)	49	.04
Per capita	0.26 (0.31)	43	0.29 (0.33)	49	.03
Per adult smoker^b^	1.67 (2.06)	43	1.93 (2.24)	49	.02
Per call^c^	101.72 (52.74)	26	103.04 (44.66)	35	.71
**Promotion**					
Total	223,553 (225,796)	34	205,891 (186,335)	41	.29
Per capita	0.12 (0.17)	34	0.14 (0.20)	41	.16
Per adult smoker^b^	0.62 (0.75)	34	0.83 (1.15)	41	.12
Per call^c^	97.38 (124.12)	20	93.61 (157.53)	31	.20

a Calculated by using McNemar χ^2^ test.

b US adults aged 18 years or older.

c Services budget divided by total calls or promotion budget divided by total calls.
